# An Account of the Plant Krameria Triandria of Linnæus;

**Published:** 1805-08-01

**Authors:** Hypolito Ruiz


An Account of the Plant Krameria Triandria of Linnæus; *extracted from a Dissertation on the Subject, first published in Vol. I. of the Memoirs of the Royal Academy of Medicine of Madrid, and republished by the Author, (in* 1799, *at Madrid,)* Don Hypolito Ruiz, *First Botanist of the Expedition to Peru, &c. &c. Communicated by* Mr. Leonard Gillespie.

THE shrub called Ratánhia by the Spanish inhabitants of the province of Huánuco in Peru, and the root of which is named the Root good for the teeth in Lenice, and the neighbouring provinces, is the Krameria foliis oblongis obovatisque, corrollis tetrapetulis, Linnæi. Flor. Peruvian. et Chil. tom. i. page 61. Icon. 93. Memor. Reg. Acad. Matrit. vol. i. p. 364.

This plant is the spontaneous growth of many provinces of Peru, delighting in a dry sandy soil, exposed to the intense heat of the vertical sun of that climate. The author of this Dissertation, discovered the plant in 1779, in the provinces of Tarma and Xauxa; in 1780 he found the same plant in the provinces of Huaorcheri and Huánuco; and in 1784 he met with it in the province of Canta, when he first became acquainted with the medicinal virtues of this simple, his curiosity having been excited by seeing some females use a species of root as a tooth brush, which on inquiry he was informed was the Ratanhia or root used for the teeth, used from time immemorial by the native Indians and Creoles, to cleanse the teeth, to fasten them, and at the same time to give a fresh colour to the gums and lips.

Don

Don Hypolito Ruiz having prepared an extract from the root of the krameria, found it to possess a degree of astringency superior to that of the tormentilla, bistorta, or alchemilla, or to that of any other simple known in the Materia Medica. He first essayed the use of this extract, in the case of a child of eleven years of age, who had been afflicted with hæmorrhage by the mouth and nose; and notwithstanding the use of the usual medicines in such cases, the flux had continued with the total prostration of the strength of the patient; a drachm of the extract dissolved in two ounces of water, with the addition of a little distilled vinegar, administered to the patient, had the effect of completely stopping the hæmorrhage, and in three days the patient was perfectly restored to health. In three obstinate cases of uterine hæmorrhage, the administration of this medicine was attended with the happiest effects. The good effects of this extract were also manifested in ulceration of the gums and mouth, in laxity of the gums, and in restraining the hæmorrhage from recent wounds, to which it was externally applied in the form of powder; and mixed with resinous substances, it was found an excellent application to ulcers in a detergent state but which were prevented from becoming cicatrized by relaxation.

The root of the Krameria was found to answer the purpose of a tooth brush extremely well, deterging the teeth, and by its powerful astringency rendering the gums firm, and of a healthy appearance. The dose of the extract generally used by the eminent physicians of Madrid, who made use of it, and whose names the author of the Dissertation makes use of in testimony of the truth of his statement, is, for a child of ten years and upwards, from one scruple to two; and in an adult the quantity of half a drachm to a drachm, dissolved in rose-water or common water, to which is added a small quantity of distilled vinegar.

The author asserts, that in some patients affected with hæmorrhage, one dose of the extract was sufficient to stop the effusion of blood; in others, the second dose had the desired effect; whilst few patients required the administration of a third dose. A decoction of the root or of the bark of the ratanhia was found to have the same effects as the extract.

The author cites, in support of the virtues attributed by him to this simple and its extract, the authorities of a long list of physicians of Madrid, who made use of this medicine, medicine, together with a list of persons of rank who had profited by the use of this remedy; and amongst the former is mentioned Dr. Don Jgnacio Ruiz de Luzuriaga, Court Physician, and well known to the writer of this article, as an experienced physician, a man of great veracity, and a Graduate of the University of Edinburgh.

The sensible qualities of the root, bark, and extract of the ratanhia certainly denote a very strong degree of astringency in this simple, and are sufficient to recommend the notice of it, as likely to prove a useful addition to the Materia Medica, although the author of the Dissertation has perhaps, like most other discoverers, raised the merits of his discovery and the virtues of this Peruvian simple far above their real value. The extract is prepared near Lenice, and imported in considerable quantities into Spain, together with the root and bark. A small quantity of each of these articles, together with the Dissertation, were lately presented to John Turnbull, Esq. Chairman of the Merchants trading to the Streights and Spain by Don Joaquin M. Ferrer, a Spanish merchant; and it is from the politeness of Mr. Turnbull, and his desire to promote the art of healing, that the present account of this Dissertation, and the new remedy it describes has had its origin.

